# Left-Sided Prosthetic Valve Dysfunction and Gastrointestinal Bleeding

**DOI:** 10.7759/cureus.37042

**Published:** 2023-04-02

**Authors:** Kifah Hussain, Ajoe J Kattoor, Bolun Liu, Agata Parfieniuk, Ikechukwu Achebe, Rami Doukky

**Affiliations:** 1 Department of Cardiology, University of Chicago (NorthShore University Health System), Chicago, USA; 2 Department of Cardiology, University at Buffalo/Kaleida Health, Buffalo, USA; 3 Department of Hospital Medicine, Mayo Clinic Health System, Mankato, USA; 4 Department of Medicine, Advocate Christ Medical Center, Oak Lawn, USA; 5 Department of Gastroenterology, University of Massachusetts Chan Medical School, Worcester, USA; 6 Department of Cardiology, John H Stroger Jr. Hospital of Cook County, Chicago, USA

**Keywords:** prosthetic valve stenosis, prosthetic valve regurgitation, prosthetic valve dysfunction, gastrointestinal tract bleeding, prosthetic heart valve

## Abstract

Introduction

We sought to investigate the association between left-sided prosthetic valve dysfunction and gastrointestinal (GI) bleeding.

Methods

In a retrospective cohort of patients with left-sided prostheses, we identified those who experienced one or more GI bleeds. The latest or chronologically closest echocardiogram to the GI bleed was analyzed by a blinded investigator for prosthetic valve dysfunction.

Results

Among 334 unique patients, 166 had aortic prostheses, 127 had mitral prostheses, and 41 had both. A total of 58 (17.4%) subjects had GI bleeding events. Patients in the *“GI Bleed” *group had higher mean ejection fraction (56±14% vs. 49±15%; P = 0.003) and higher prevalence of hypertension, end-stage renal disease, and liver cirrhosis compared to the *“No GI Bleed” *group. There was a higher prevalence of moderate or severe prosthetic valve regurgitation in the GI Bleed vs. No GI Bleed group (8.6% vs. 2.2%; P = 0.027). Moderate or severe prosthetic valve regurgitation was independently associated with GI bleeding (odds ratio, 6.18; 95% confidence interval, 1.27-30.05; P = 0.024), after adjusting for ejection fraction, hypertension, end-stage renal disease and liver cirrhosis. Paravalvular regurgitation was associated with a higher incidence of GI bleeding compared to transvalvular regurgitation (35.7% vs. 11.9%; P = 0.044). The prevalence of prosthetic valve stenosis was similar between the GI Bleed and No GI Bleed groups (6.9% vs. 5.8%; P = 0.761).

Conclusion

In a cohort of patients with predominantly surgically placed prosthetic valves, moderate to severe left-sided prosthetic valve regurgitation was independently associated with GI bleeding.

## Introduction

Prosthetic valve (PV) dysfunction can occur either from intrinsic structural valve deterioration or non-structural dysfunction, such as entrapment by pannus or tissue, thrombosis, paravalvular leak, or severe patient prosthetic mismatch [1]. Prior studies have reported a possible increased incidence of gastrointestinal (GI) bleeding in patients with aortic and mitral PV dysfunction. These investigations have postulated a mechanism similar to that of Heyde’s syndrome, wherein von Willebrand Factor (VWF) multimers are broken down as a result of the shear stress, resulting in angiodysplasia-induced GI bleeding [2-4]. Studies have also suggested that paravalvular regurgitation may be an independent predictor of GI bleeding following transcatheter aortic valve replacement (TAVR) [5, 6]. However, data on surgically placed prosthetic valves is limited. We sought to investigate a hypothesized association between left-sided prosthetic valve dysfunction and GI bleeding.

## Materials and methods

Study population and clinical data

In a retrospective cohort study design, all patients over 18 years of age, who had transthoracic or transesophageal echocardiograms performed in the period from June 1, 2018 to March 31, 2020 were identified using the electronic database of Cook County Health (Chicago, Illinois, USA). Those whose echocardiogram reports suggested the presence of either mechanical or bioprosthetic prostheses, in the aortic or mitral positions were included in the study. The rationale for including patients with surgical or transcatheter mitral valve repair in our cohort is that the data indicates that there is an association between these types of repairs and GI bleeding events and acquired von Willebrand disease [7-9]. Electronic medical records of these patients were reviewed for demographic, clinical, laboratory, and echocardiographic data. Based on the occurrence of GI bleeding events after PV implantation, patients were categorized into “GI Bleed” and “No GI Bleed” study groups. GI bleeding events were defined using a combination of clinical and laboratory data to include patients who had one or more episodes of melena, hematochezia, hematemesis, and/or had evidence of iron deficiency anemia secondary to GI bleeding. For patients in the GI Bleed group, laboratory parameters on the day of the GI bleed or immediately prior to it were selected. For patients in the No GI Bleed group, the laboratory values closest to an index echocardiogram were recorded.

Echocardiographic assessment

An index echocardiogram was identified for analysis. For patients with a GI bleed, the index echocardiogram was the one chronologically closest to the GI bleed (before or after), whereas for those without a GI bleed, the latest available echocardiogram was selected for review. An experienced reader blinded to clinical data and GI bleeding status (AJK) reviewed all echocardiographic images to assess prosthetic valve function according to the 2009 American Society of Echocardiography guidelines for the evaluation of prosthetic valves [10]. Left ventricular ejection fraction (LVEF) was assessed using the modified Simpson's method. Two-dimensional guided left ventricular linear measurements were used for the assessment of left ventricular dilation based on the 2015 American Society of Echocardiography guidelines on Cardiac Chamber Quantification [11]. The primary echocardiographic assessment was based on transthoracic echocardiograms; however, when available, transesophageal echocardiograms were also reviewed to improve accuracy in the assessment of PV dysfunction. Mitral valve repair with annuloplasty ring, mitral clip, mitral valve-in-valve, and transcatheter aortic valves, were considered as bioprosthetic valves for the study purposes.

Statistical analyses

Data were expressed as numbers and percentages for categorical variables and means ± standard deviations or median with interquartile range for continuous variables. As appropriate, we used the chi-square test or the Fisher’s exact test to compare categorical variables and the independent samples Student’s t-test or the Mann-Whitney test to compare continuous variables. Multivariate logistic regression models were used to determine factors independently associated with GI bleeding, adjusting for covariates shown to be associated with GI bleeding in univariate analyses. To avoid overfitting the logistic regression models, covariates were carefully chosen not to exceed an approximate ratio of 1 degree of freedom for every 10 GI bleeding events. SPSS version 27 (IBM Corp., Armonk, NY) software package was used for all statistical analyses. The study was approved by the Institutional Review Board of Cook County Health.

This article was previously posted to the Authorea preprint server on April 05, 2022.

## Results

Database query yielded 334 unique patients with left-sided PV, among those 166 had aortic prostheses, 127 had mitral prostheses, and 41 had both aortic and mitral prostheses. There were 86 patients with a mechanical aortic prosthesis and 121 with a bioprosthetic aortic prosthesis, of which three patients had TAVR. There were 81 patients with a mechanical mitral prosthesis and 87 with a bioprosthetic mitral prosthesis, among whom three had mitral clips and one had a transcatheter valve-in-valve implant. The mean age was 60 ± 13 years and 178 (53.3%) were men. The mean age of patients at the time of their valvular intervention was 52 ± 15 years. A total of 58 (17.4%) subjects had at least one episode of GI bleeding, among whom 34 had aortic prostheses, 18 had mitral prostheses, and six had both aortic and mitral prostheses.

GI bleeding

Among the 58 patients with GI bleed, 19 (32.8%) presented with melena, 18 (31.0%) with hematochezia, four (6.9%) with hematemesis, 13 (22.4%) with anemia (iron deficiency or presumed acute blood loss), and four (6.9%) had non-specific symptoms but were found to have GI bleeding on endoscopy. Seven (12.1%) patients had major GI bleeding, defined as requiring a transfusion of ≥4 packed red blood cells, 18 (31.0%) required <4 packed red blood cells, while 33 (56.9%) did not require any blood transfusion. Twenty-two (37.9%) patients were found to have an obvious source of bleeding on endoscopy, while 36 (62.1%) had obscure bleeding. The median duration from valve intervention to GI bleeding event was 51 months (25th-75th percentile, 8.5-151).

The baseline characteristics of the GI Bleed and No GI Bleed study groups are summarized in Table 1. Notably, hypertension, end-stage renal disease (ESRD), and liver cirrhosis were significantly more prevalent among patients with GI bleed. There was no statistically significant difference in the prevalence of antiplatelet and anticoagulant use between the study groups. Expectedly, the mean hemoglobin level was significantly lower among patients in the GI Bleed group. Platelet count, international normalized ratio (INR), were similar in both groups, but the mean serum creatinine was higher in the GI Bleed group.

**Table 1:** Baseline Characteristics Values are depicted as means ± standard deviations for continuous variables and numbers (%) for dichotomous variables. ESRD: End-stage renal disease; GI: Gastrointestinal; INR: International normalized ratio

Variable	GI Bleed	No GI Bleed	P value
All Subjects (N, 334)	N, 58	N, 276	
Age at the time of echocardiogram	60 ± 12	60 ± 13	0.711
Male sex	32 (55.2)	146 (52.9)	0.752
BMI	29.7 ± 9.0	29.4 ± 7.2	0.692
Ethnicity			0.486
African American	103 (37.3)	23 (39.7)	
Hispanic	89 (32.2)	23 (39.7)	
White	55 (19.9)	8 (13.8)	
Others	4 (6.9)	29 (10.5)	
Hypertension	49 (84.5)	199 (72.1)	0.049
Diabetes	19 (32.8)	75 (27.2)	0.390
Heart failure	21 (36.2)	117 (42.4)	0.385
Stroke	6 (10.3)	38 (13.8)	0.483
ESRD	5 (8.6)	3 (1.1)	0.005
Liver cirrhosis	9 (15.5)	9 (3.3)	<0.001
Oral anticoagulant use	39 (67.3)	183 (66.3)	0.919
Antiplatelets use	33 (56.9)	192 (69.6)	0.061
Hemoglobin (g/dL)	10 ± 3	12 ± 2	<0.001
Platelet count (x 10^3^)	220 ± 75	219 ± 83	0.914
INR	2.3 ± 1.7	2.1 ± 1.2	0.238
Serum creatinine (mg/dL)	1.7 ± 2.0	1.1 ± 0.6	0.022

Echocardiographic findings

**Figure 1:**
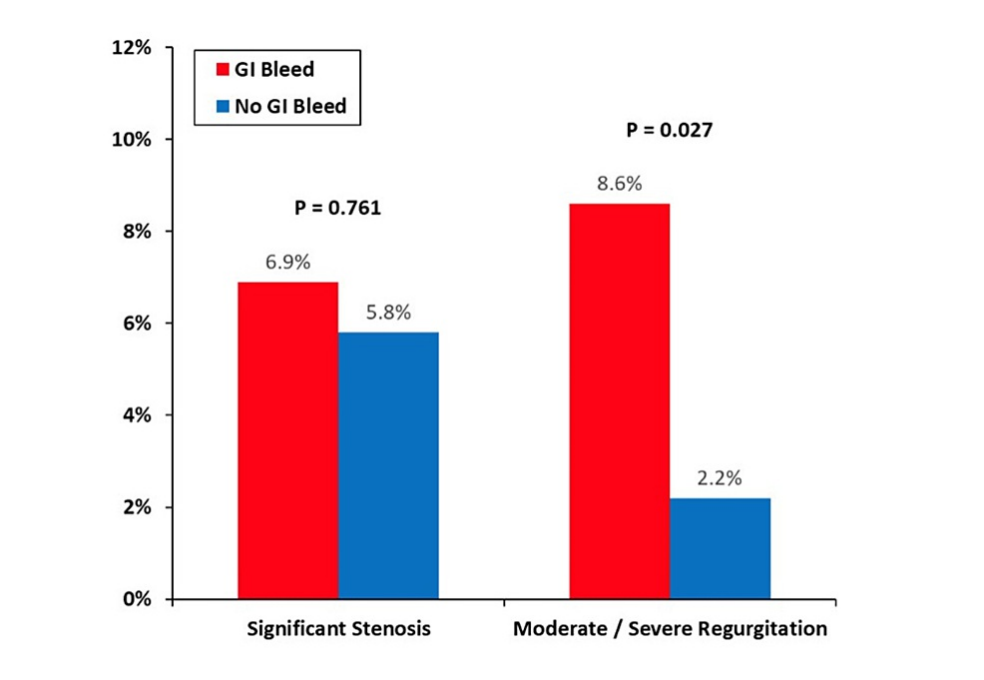
Prosthetic Valve Dysfunction and Gastrointestinal Bleeding.

Table 2 summarizes the echocardiographic findings in the study groups. Notably, patients in the GI Bleed group had higher mean LVEF but similar prevalence of left ventricular dilation. As shown in Figure 1, there was no significant difference in the prevalence of significant PV stenosis (6.9% vs. 5.8%; P = 0.761) or PV regurgitation of any degree (mild, moderate, or severe) (17.2% vs. 16.7%; P = 0.915) between patients with and without GI bleeding. However, there was a higher prevalence of moderate or severe PV regurgitation in the GI Bleed group compared to the No GI Bleed group (8.6% vs. 2.2%; P = 0.027). There was a statistically insignificant increase in the rate of GI bleeding among patients with paravalvular regurgitation (Table 2).

**Table 2:** Echocardiographic Findings Values are depicted as means ± standard deviations for continuous variables and numbers (%) for dichotomous variables. * 41 subjects had mitral and aortic prostheses EOA: Effective Orifice Area; GI: Gastrointestinal; PV: Prosthetic valve

Variable	GI Bleed	No GI Bleed	P value
All Subjects (N, 334)	N, 58	N, 276	
Ejection fraction (%)	56 ± 14	49 ± 15	0.002
LV dilation	8 (13.8)	50 (18.2)	0.423
Any degree of PV regurgitation	10 (17.2)	46 (16.7)	0.915
Paravalvular regurgitation	5 (8.6%)	9 (3.3%)	0.064
Moderate/severe PV regurgitation	5 (8.6)	6 (2.2)	0.027
Significant PV stenosis	4 (6.9)	16 (5.8)	0.761
Mitral prosthesis (N, 168)*	N, 24	N, 144	
Prosthesis type			0.488
Bioprosthetic	14 (58.3)	73 (50.7)	
Mechanical	10 (41.7)	71 (49.3)	
Vmax, m/s	1.9 ± 0.4	1.6 ± 0.4	0.014
Mean gradient, mmHg	8.9 ± 15.8	4.5 ± 2.5	0.002
Pressure half time, ms	93 ± 33	97 ± 41	0.718
Dimensionless Index	1.8 ± 0.8	2.1 ± 0.9	0.282
EOA, cm^2^	1.8 ± 0.6	1.7 ± 0.8	0.906
Moderate/severe mitral regurgitation	3 (12.5)	5 (3.5)	0.089
Significant mitral stenosis	3 (12.5)	7 (4.9)	0.156
Aortic prosthesis (N, 207)*	N, 40	N, 167	
Prosthesis type			0.622
Bioprosthetic	22 (55.0)	99 (59.3)	
Mechanical	18 (45.0)	68 (40.7)	
Vmax, m/s	2.7 ± 0.8	2.6 ± 0.7	0.544
Mean gradient, mmHg	16.7 ± 10.3	16.1 ± 9.3	0.713
Acceleration time, ms	76 ± 14	80 ± 15	0.087
Dimensionless index	0.5 ± 0.1	0.5 ± 0.2	0.989
EOA, cm^2^	1.5 ± 0.6	1.5 ± 0.6	0.867
Moderate/severe aortic regurgitation	2 (5.0)	1 (0.6)	0.096
Significant aortic stenosis	1 (2.5)	9 (5.4)	0.691

Among 56 subjects with PV regurgitation of any degree, paravalvular regurgitation was associated with a higher incidence of GI bleeding than transvalvular regurgitation [5/14 (35.7%) vs. 5/42 (11.9%); P = 0.044]. In exploratory analysis of 11 subjects with moderate or severe PV regurgitation, the incidence of GI bleeding was numerically higher among patients with paravalvular regurgitation than among those with transvalvular regurgitation [2/2 (100.0%) vs. 3/9 (33.3%); Fisher’s exact P = 0.182].

In multivariate logistic regression analysis, adjusting for hypertension, liver cirrhosis, ESRD, and LVEF, moderate or severe left-sided PV regurgitation was independently associated with GI bleeding (OR, 6.18; 95% confidence interval [CI], 1.27-30.05; P = 0.024). In this model, liver cirrhosis (OR, 7.66; 95% CI, 2.50-23.49; P < 0.001), ESRD (OR, 6.06; 95% CI, 1.21-30.24; P = 0.028) and LVEF (OR, 1.48 per 10% point increase; 95% CI, 1.17-1.89; P = 0.001) were independently associated with GI bleeding, whereas hypertension was not (OR, 1.71; 95% CI, 0.78-3.77; P = 0.184) (Table 3). The association between moderate or severe PV regurgitation and GI bleeding was consistent irrespective of the valve position - aortic or mitral (interaction P = 0.261) or valve type - mechanical vs. bioprosthetic (interaction P = 0.999).

**Table 3:** Logistic multivariate regression analysis for the association of left-sided prosthetic valve regurgitation and gastrointestinal bleeding ESRD: End-stage renal disease; GI: Gastrointestinal; LVEF: Left ventricular ejection fraction

Variables	Odds ratio	(95% confidence interval)	p-value
ESRD	6.06	1.21 - 30.24	0.028
Hypertension	1.71	0.78 - 3.77	0.184
Liver cirrhosis	7.66	2.50 - 23.49	< 0.001
LVEF	1.48	1.17 - 1.89	0.001

Subgroup analyses

The study is underpowered to detect meaningful differences between patients with GI bleeding vs. those with no GI bleeding in subgroups of mitral and aortic prosthesis. However, we performed these analyses to ensure that the data trends in these subgroups are consistent with the entire study population.

In subgroups of patients with aortic or mitral prostheses, there was a trend towards a higher prevalence of moderate or severe prosthetic aortic valve regurgitation (5.0% vs. 0.6%; P = 0.096) and moderate or severe prosthetic mitral valve regurgitation (12.5% vs. 3.5%; P = 0.089) among patients with GI bleeding vs. those with no GI bleeding. On the other hand, there was no significant difference in the prevalence of significant PV stenosis, in the aortic or mitral position, among patients with GI bleeding vs. those with no GI bleeding (Table 2). Regardless of prosthesis position, there was no significant difference in the mean effective orifice area between those with and without GI bleeding. Furthermore, regardless of the valve position, there was no significant difference in the prevalence of bioprosthetic vs. mechanical prostheses among patients with GI bleed vs. no GI bleed (Table 2).

In the mitral prosthesis subgroup, patients with a GI bleed had significantly higher mitral valve peak velocity and mean gradient compared to those with no GI bleed (Table 2). However, these associations between increased transvalvular velocity (OR, 3.54 per 1 m/s; 95% CI, 0.99-12.66; P = 0.052) and gradient (OR, 1.14 per 1 mmHg; 95% CI, 0.98-1.32; P = 0.083) were found not to be statistically significant after adjusting for hemoglobin level and moderate or severe PV regurgitation status. This suggests that the increase in velocity and gradient are in part driven by increased flow due to anemia or regurgitation.

## Discussion

To the best of our knowledge, this is the first study to investigate the association between prosthetic valve dysfunction and GI bleeding in a cohort of predominantly surgically implanted valves. We demonstrated that moderate or severe left-sided PV regurgitation is associated with an increased incidence of GI bleeding. This association was independent of clinical covariates and was consistent regardless of valve position (aortic vs. mitral) and type (mechanical vs. bioprosthetic). In exploratory analysis, it seems that the incidence of GI bleeding is greater among subjects with paravalvular compared to transvalvular regurgitation. This observation needs to be validated in future research. On the other hand, there was no significant association between PV stenosis and GI bleeding. Liver cirrhosis, ESRD, and higher LVEF were independently associated with GI bleeding in this population.

There is a paucity of data regarding the association between PV dysfunction and GI bleeding. In a study of 136 patients, Blackshear et al. demonstrated the prevalence of abnormal VWF was significantly higher among patients with PV dysfunction than those with normally functioning prostheses (79.1% vs. 4.8%) [2]. The authors also noted an increase in GI bleeding in the PV dysfunction group. However, the study’s small sample size did not allow for the adjustment of clinical or echocardiographic covariates. Vincentelli et al. studied the effect of valve replacement on VWF activity in patients with native aortic valve stenosis. They reported an association between patient-prosthesis mismatch and lack of normalization of VWF multimers following surgical aortic valve replacement, suggesting that increased shear stress may be associated with acquired VWF abnormality [12]. Van Belle et al. studied the recovery of VWF multimers following TAVR in 20 patients with aortic stenosis. Recovery of high molecular weight multimers occurred within minutes of acute drop in shear stress following implantation of TAVR valve. However, no recovery of VWF was observed in four patients with residual aortic regurgitation [13].

The association between paravalvular regurgitation and GI bleeding has been described in TAVR patients. In an analysis of the PARTNER registry, Généreux et al. reported that the presence of moderate or severe paravalvular regurgitation at 30 days was an independent predictor of major late (>30 days) bleeding complications after TAVR. GI bleeding accounted for 40.8% of these events [6]. Similarly, in a cohort of 372 patients, Kibler et al. reported that paravalvular regurgitation following TAVR was independently associated with major late bleeding complications (HR, 6.3), and GI bleeding contributed to 42.8% of these events [5]. Findings from our cohort, with predominantly surgically implanted PV, are consistent with the prevailing literature. As is the case with paravalvular regurgitation in patients with TAVR, moderate or severe regurgitation in patients with surgically implanted valves is associated with higher rates of GI bleeding. Our study also suggests that paravalvular regurgitation has a greater association with GI bleeding, in agreement with TAVR data. Notably, only three patients in our cohort had TAVR, none of whom had valvular dysfunction or GI bleeding. A prior study has demonstrated a relationship between isolated mitral valve surgery including both mitral valve repair and replacement, and gastrointestinal bleeding [7]. A similar association of gastrointestinal bleeding has been reported in over 30% of patients after the implantation of MitraClip and it has been found to be the second most common cause of bleeding complications post-procedurally following access site-related bleeding [8, 9].

We postulate that VWF abnormality is the likely mechanism behind our findings. As in the case of severe native aortic stenosis, high shear stress is proposed to cause changes in the morphology of von Willebrand multimers, making them more susceptible to cleavage by ADAMTS13 (the von Willebrand multimer cleaving metalloprotease) and thereby, causing an acquired abnormality in hemostasis [4, 14]. There are reports of moderate or severe native mitral regurgitation to be associated with abnormal VWF multimers and clinically significant GI bleeding, which reversed after mitral valve surgery [15]. In 123 patients with moderate or severe native mitral regurgitation undergoing transcatheter mitral valve repair, Meindl et al. reported that an elevated mean mitral valve gradient following transcatheter mitral valve repair was associated with a low post-procedure VWF activity [16]. However, this alteration did not translate into a higher incidence of bleeding events. We can speculate that the restrictive flow in transvalvular or paravalvular prosthetic valve regurgitation may contribute to acquired von Willebrand disease resulting in GI bleeding, similar to that seen in native aortic stenosis [3, 4, 14].

Notably, we were unable to demonstrate a statistically significant association between prosthetic valve stenosis and GI bleeding. This is likely related to a relatively small sample size. The effect size of moderate or severe regurgitation on GI bleeding (OR = 6.18) seems to be stronger than that of PV stenosis (OR = 1.20), an observation that can be explained by the fact that, in most cases, PV regurgitation causes more restrictive flow environment than PV stenosis. More studies are needed to ascertain a cause-effect relationship between PV dysfunction, VWF abnormality, and GI bleeding. Future research needs to determine whether risk for GI bleeding can be reduced with valve replacement or treatment of paravalvular regurgitation using transcatheter procedures.

It is notable that GI bleeding is a major comorbidity in patients with PV implants. Nearly 17.4% of patients in our cohort had at least one episode of GI bleeding at some point after valve implantation. The incidence of GI bleeding events was comparable in patients with bioprosthetic and mechanical valves despite oral anticoagulation use with the latter. Furthermore, the prevalence of oral anticoagulant use was comparable in GI Bleed and No GI Bleed groups.

Higher LVEF was independently associated with GI bleeding in our cohort. We postulate that higher LVEF may lead to higher shear stress across the prosthetic valve, thereby contributing to an increase in acquired abnormality of VWF multimers and subsequent GI bleeding. In contrast, LVEF was not significantly different in patients with major late bleeding complications in the PARTNER trial data [6]. In addition, ESRD and liver cirrhosis were associated with GI bleeding. In prior studies, hypertension has been reported to be independently associated with GI bleeding from GI angiodysplasias [17, 18]. Nakatsu et al. reported that major bleeding complications are common after prosthetic valve placement in ESRD patients [19]. Multiple studies have demonstrated that patients with chronic kidney disease have low levels of VWF multimers [20-22]. Similarly, few studies have shown that patients with liver cirrhosis have lower levels of high molecular weight VWF multimers [23, 24]. We propose that increased cardiac output associated with ESRD, due to dialysis arteriovenous fistula, and liver cirrhosis, due to peripheral vasodilation, may result in increased shear stress across dysfunctional PV, leading to an abnormality in VWF multimers and GI bleeding [25, 26]. Clearly, ESRD and liver cirrhosis are in themselves causes for bleeding diathesis.

Strengths and limitation

The availability of detailed patient-level clinical data and blinded interpretation of echocardiographic images are among the strengths of our study. On the other hand, the single-center, observational study design, and relatively small sample size are clear limitations. We also do not have the data on patients with history of GI bleed prior to valve implantation. The lack of VWF function data to establish probable mechanistic correlation is another limitation.

## Conclusions

GI bleeding has a high prevalence in patients who have undergone prosthetic valve intervention. Given the association of moderate to severe left-sided prosthetic valve regurgitation and GI bleeding, an occurrence of the same should prompt discussions regarding the role of repeat valvular intervention. Furthermore, GI bleeding has an even stronger correlation with paravalvular regurgitation and warrants consideration as a potential indication to repair paravalvular leaks. The presence of additional risk enhancers including a higher LVEF, ESRD, and/or liver cirrhosis should also be highlighted during valve team discussions for these patients. The findings from this study should prompt further research into echocardiographic findings in left-sided valvular dysfunction that may have an increased association with GI bleeding and, therefore, warrant earlier management. Similarly, further studies of this correlation in predominantly percutaneously implanted or repaired valves should also be undertaken.
